# A Review on the Crosstalk between Insulin and Wnt/β-Catenin Signalling for Bone Health

**DOI:** 10.3390/ijms241512441

**Published:** 2023-08-04

**Authors:** Sok Kuan Wong, Nur Vaizura Mohamad, Putri Ayu Jayusman, Nurul ‘Izzah Ibrahim

**Affiliations:** 1 Department of Pharmacology, Faculty of Medicine, Universiti Kebangsaan Malaysia, Jalan Yaacob Latif, Bandar Tun Razak, Cheras, Kuala Lumpur 56000, Malaysia; nurulizzah@ukm.edu.my; 2 Centre for Drug and Herbal Development, Faculty of Pharmacy, Universiti Kebangsaan Malaysia, Jalan Raja Muda Abdul Aziz, Kuala Lumpur 50300, Malaysia; vaizura@ukm.edu.my; 3 Department of Craniofacial Diagnostics and Biosciences, Faculty of Dentistry, Universiti Kebangsaan Malaysia, Jalan Raja Muda Abdul Aziz, Kuala Lumpur 50300, Malaysia; putri.ayujay@gmail.com

**Keywords:** bone loss, glycogen synthase kinase-3 beta, GSK3β, type 1 diabetes mellitus, type 2 diabetes mellitus

## Abstract

A positive association between insulin resistance and osteoporosis has been widely established. However, crosstalk between the signalling molecules in insulin and Wingless (Wnt)/beta-(β-)catenin transduction cascades orchestrating bone homeostasis remains not well understood. The current review aims to collate the existing evidence, reporting (a) the expression of insulin signalling molecules involved in bone-related disorders and (b) the expression of Wnt/β-catenin signalling molecules involved in governing insulin homeostasis. The downstream effector molecule, glycogen synthase kinase-3 beta (GSK3β), has been identified to be a point of convergence linking the two signal transduction networks. This review highlights that GSK3β may be a drug target in the development of novel anabolic agents and the potential use of GSK3β inhibitors to treat bone-related disorders.

## 1. Introduction

Bone is a dynamic organ continuously undergoing bone resorption by osteoclasts and bone formation by osteoblasts to maintain bone health. The imbalance between the two concerted processes leads to the development and progression of osteoporosis. These processes are tightly regulated by several local and systemic factors such as hormones, cytokines, chemokines growth factors, and exogenous biomechanical stimulation [1]. Insulin and insulin-like growth factor-1 (IGF-1) are hormonal factors that determine bone mass variability due to the distribution of respective receptors in bone cells [2,3]. They are similar in molecular structure but differ in the production site. Insulin is produced by the β-cells of the pancreas in response to glucose, whereas IGF-1 is synthesised by the liver in a pituitary gland-derived growth hormone-dependent manner.

Circulating insulin and IGF-1 to osteoblasts exerts an anabolic signal and promotes bone formation. An inverse relationship between glycated haemoglobin, insulin level, and insulin resistance with the trabecular bone score was reported in non-diabetic postmenopausal women [4]. In another study, a positive association was observed between insulin resistance and osteoporosis, whereas a negative relationship was noted between β-cell function and osteoporosis among Taiwanese participants [5]. In patients with type 2 diabetes mellitus (T2DM), those receiving oral glucose-lowering medication and long-acting insulin displayed higher spine bone mineral density (BMD) and serum calcium levels compared to those receiving oral glucose-lowering medication only [6]. Rats fed with a high-fat, high-carbohydrate diet displayed insulin resistance and a deterioration of the trabecular bone microstructure [7,8,9,10,11]. Insulin exerts anabolic action on bone, whereby high insulin and low glucose enhance osteocalcin (OCN) production but disrupted insulin signalling and high glucose decreases OCN production [12]. Higher serum IGF-1 was associated with greater BMD and reduced fracture risk in European men and women [13]. In animals, the deficiency of IGF-1 early in life resulted in pronounced reductions in cortical bone fraction and thickness [14]. In addition, bone is an endocrine organ that secretes OCN, which is responsible for several physiological processes including the proliferation of β-cells, regulation of insulin secretion and sensitivity, and regulation of energy and glucose metabolism [15].

Canonical Wingless (Wnt)/beta-(β-)catenin signalling has a central role in the maintenance of bone homeostasis by regulating the osteogenic differentiation of mesenchymal stem cells, the proliferation of osteoblasts, as well as the activation and maturation of osteoclasts [16]. Wnt ligands trigger the intracellular signalling cascade to accumulate β-catenin in bone microenvironments upon binding to the co-receptors, suggesting the significant contribution of Wnt ligands, co-receptors, and inhibitors in skeletal phenotypes in experimental and clinical settings. The signalling crosstalk between other regulatory networks (such as the non-canonical Wnt, Janus kinase (JAK)/signal transducers and activators of transcription (STAT), as well as Hedgehog pathways) with the canonical Wnt/β-catenin pathway synergistically achieves the regulation of bone metabolism. In addition to the osteogenic properties, the presence of the Wnt agonist stimulated glucose uptake and ameliorated insulin resistance via an increased expression of insulin receptor substrate (IRS)-1 in rat primary neurons [17].

Current knowledge focusing on the role of insulin in bone health and the role of Wnt ligands in insulin sensitivity has been established; however, the crosstalk between the insulin and Wnt/β-catenin signalling molecules remains not fully understood. Herein, the current review aims to summarise the documented evidence on the effects of insulin and/or IGF-1 on the Wnt/β-catenin pathway as well as the effects of Wnt signalling molecules on insulin secretion and/or sensitivity. Reviewing the literature helps to provide a comprehensive overview of the possible underlying molecule that acts as the molecular link responsible for the existing intricacy between the two signalling pathways. The understanding of this aspect allows future advancement in the identification of promising drug targets for the management of insulin- and/or skeletal-related disturbances.

## 2. Literature Search

**Figure 1:**
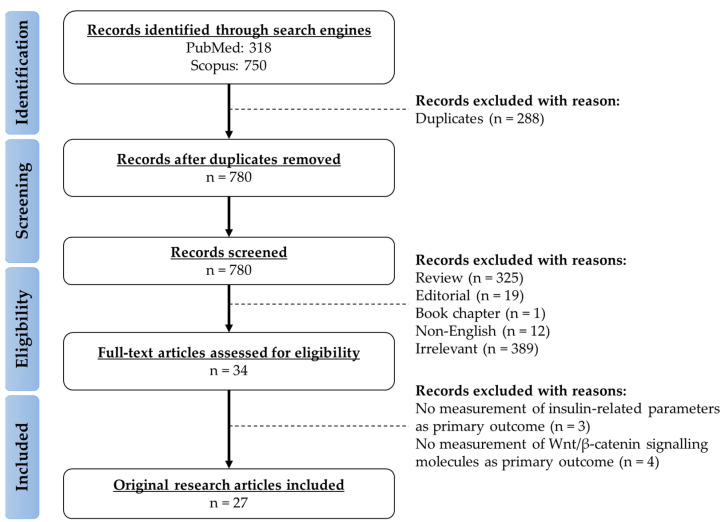
Framework of literature search.

The literature acquisition was performed using two electronic databases (PubMed and Scopus) in April 2023. The search string used for the literature search was “(insulin OR “insulin receptor” OR “insulin-like growth factor-1” OR IGF-1) AND (Wnt OR beta-catenin) AND (bone OR osteoporosis OR fracture OR osteoblast OR osteoclast OR osteocyte)”. All records from the inception of the databases were searched. A total of 318 and 750 records were obtained from PubMed and Scopus databases, respectively. Duplicates (n = 288) were removed. The title of the remaining records was screened to remove reviews (n = 325), editorials (n = 19), book chapters (n = 1), non-English articles (n = 12), and irrelevant articles (n = 389). From the remaining records, full-text articles were assessed for eligibility, and a total of 27 articles were included based on the inclusion criteria. The current review includes all original research articles reporting both the insulin- and IGF-1-related parameters as well as the Wnt-related signalling molecules as primary outcomes using human subjects, animals, and cell culture as experimental models. Studies were excluded if they only met one of the aforementioned aspects (i.e., insulin-/IGF-1-related parameters or Wnt-related signalling molecules) (Figure 1).

## 3. The Insulin and IGF-1 Signalling Pathway

Insulin is an essential hormone that functions in controlling blood glucose levels by regulating the metabolism of macronutrients (carbohydrate, lipid, and protein) and facilitating cellular glucose transport [18]. On the other hand, IGF-1 is a peptide that promotes cell growth, proliferation, and maturation. It binds to the insulin-like growth factor binding protein (IGFBP), which acts as the carrier to stabilise IGF-1 and modulates the interaction between IGF-1 and its receptor [19]. The equal or greater affinity of binding between IGF-1 and IGFBP than IGF-1 and IGF-1 receptor (IGF-1R) suggests the role of IGFBP in regulating IGF-1 signalling. For instance, the binding of IGF-1 to IGFBP sequesters IGF-1 from the receptors and inhibits IGF-1 signalling. The presence of protease to cleave IGFBP in peripheral tissues leads to the release of IGF-1 and increases its availability towards IGF-1R. In addition, certain IGFBPs bind to proteoglycans on the cell surface, resulting in the liberation of IGF-1 to IGF-1R [20].

**Figure 2:**
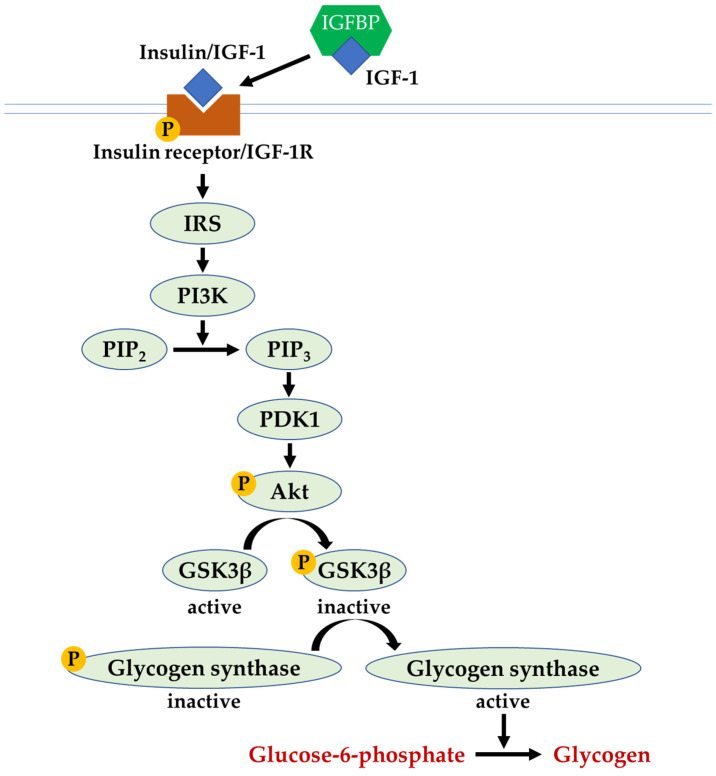
The insulin and IGF-1 signalling pathway. Abbreviations: Akt, protein kinase B; GSK3β, glycogen synthase kinase-3 beta; IGF-1, insulin-like growth factor-1; IGFBP, insulin-like growth factor binding protein; IRS, insulin receptor substrate; p-Akt, phosphorylated protein kinase B; PDK1, phosphoinositide-dependent kinase-1; p-glycogen synthase, phosphorylated glycogen synthase; p-GSK3β, phosphorylated glycogen synthase kinase-3 beta; PI3K, phosphoinositide-3-kinases; p-IGF-1R, phosphorylated insulin-like growth factor-1 receptor; p-insulin receptor, phosphorylated insulin receptor; PIP_2_, phosphatidylinositol-4,5-biphosphate; PIP_3_, phosphatidylinositol-3,4,5-triphosphate.

Apart from binding its own receptor, both insulin and IGF-1 bind and activate each other’s receptors with reduced affinity. In the insulin and IGF-1 signalling pathway, the binding of insulin or IGF-1 to their receptors (insulin receptor and/or IGF-1R) triggers the autophosphorylation of the receptors, generating the binding site for IRS-1 and causing its phosphorylation (activation). The activated IRS-1 binds and activates phosphoinositide-3-kinases (PI3K). Next, PI3K catalyses the conversion of phosphatidylinositol-4,5-biphosphate (PIP_2_) to phosphatidylinositol-3,4,5-triphosphate (PIP_3_), which, in turn, induces a cascade of kinase activity through phosphoinositide-dependent kinase-1 (PDK1) and protein kinase B (Akt). The activation of Akt phosphorylates glycogen synthase kinase-3 beta (GSK3β) causes its inactivation, which subsequently results in glycogen synthase activation and the synthesis of glycogen in the liver cells. In the state of insulin resistance, the inhibition of downstream insulin signal transduction activates GSK3β; thus, glycogen production is attenuated [21] (Figure 2).

## 4. The Wnt/β-Catenin Signalling Pathway

**Figure 3:**
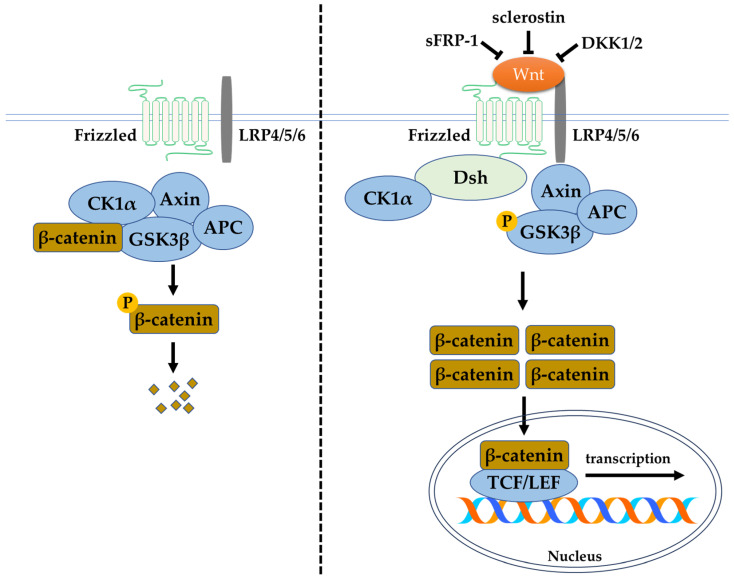
The Wnt/β-catenin signalling pathway. Abbreviations: Axin, axis inhibition protein; APC, adenomatosis polyposis coli; CK1α, casein kinase 1α; DKK1, Dickkopf-related protein 1; DKK2, Dickkopf-related protein 2; Dsh, phosphoprotein Dishevelled; GSK3β, glycogen synthase kinase-3 beta; LEF, lymphoid enhancing factor; LRP4/5/6, lipoprotein receptor-related protein 4/5/6; p-β-catenin, phosphorylated β-catenin; p-GSK3β, phosphorylated glycogen synthase kinase-3 beta; sFRP-1, secreted frizzled-related protein-1; TCF, T-cell factor.

The canonical Wnt/β-catenin signalling pathway depends on the function of β-catenin, which is the central and critical molecule to this pathway. Various receptors, inhibitors, activators, modulators, phosphatases, and kinases are involved in this signalling pathway. The receptors (such as lipoprotein receptor-related protein (LRP) 4/5/6)), activators (such as the Wnt ligands), and inhibitors (including secreted frizzled-related protein (sFRP)-1, sclerostin, and Dickkopf-related protein 1 (DKK1)) modulate the phosphorylation of β-catenin. These regulators or modulators target β-catenin by causing its phosphorylation and degradation via proteasomes or by causing its non-phosphorylation and regulation of gene expression in the nucleus. Without Wnt ligand in the canonical Wnt/β-catenin signalling pathway, the presence of the destruction complex consisting of an axis inhibition protein (Axin), adenomatosis polyposis coli (APC), protein phosphatase 2A (PP2A), GSK3β, and casein kinase 1α (CK1α) causes the ubiquitination and degradation of β-catenin. Thus, the accumulation of β-catenin in the cytoplasm, and the eventual translocation into the nucleus for T-cell factor/lymphoid-enhancing factor (TCF/LEF) family transcriptional activity, does not occur. Likewise, the presence of Wnt inhibitors inactivates the canonical Wnt/β-catenin pathway and inhibits bone formation. High circulating levels of sclerostin and DKK1 have been associated with clinical bone pathologies, such as bone pain, structural bone changes, osteoporosis, and bone deformity. Thus, the circulated level of Wnt inhibitors are potential biomarkers of skeletal abnormalities [22]. In the absence of inhibitory control, the activation of the Wnt/β-catenin signalling pathway begins with the binding of the Wnt ligand to the membrane-bound frizzled family receptor and LRP4/5/6 to initiate a cellular response. Upon frizzled family receptor activation, a signal is sent to the phosphoprotein Dishevelled (Dsh) via direct interaction and causes its activation. The activated Dsh inhibits GSK3β, leading to the disruption of the destruction complex. This allows the accumulation and translocation of β-catenin into the nucleus for the expression of downstream transcription factors [23]. In the bone tissue, this pathway assists in the maintenance of physiological bone remodelling. Activation and inhibition of the pathway promote bone formation and resorption, respectively (Figure 3).

## 5. The Crosstalk between Insulin and Wnt/β-Catenin Signalling in Human Studies

Previous evidence has shown the association between insulin and insulin-related components with bone-related Wnt/β-catenin signalling pathways in human studies (Table 1). In a case-control study by Kurban et al., they demonstrated that insulin and DKK1 levels were higher in children with type 1 diabetes mellitus (T1DM) than in controls. Several parameters related to bone such as N-terminal telopeptide (NTX), osteocalcin (OCN), vitamin D, and phosphorus were reduced in children with T1DM, indicating lower bone remodelling and its compensatory mechanism for bone loss [24]. The decreased circulating biochemical markers of bone turnover have been associated with diabetes mellitus and could be predictive of fractures independently of BMD [25]. Similar outcomes were observed in another cross-sectional study, whereby T1DM children and adolescents receiving insulin treatment had higher levels of DKK1 and sclerostin but a lower Z-score than the controls [26]. The T1DM children and adolescents received either multiple daily injections or a continuous subcutaneous infusion of insulin. Between the two experimental approaches, improvements in glycaemic control and Z-score were seen in the T1DM children and adolescents after receiving the continuous subcutaneous infusion of insulin compared to those receiving multiple daily injections of insulin [26]. In a study by Razny et al., DKK1, along with other Wnt signalling inhibitors such as Dickkopf-related protein 2 (DKK2), sclerostin, and sFRP-1 levels, was increased in obese patients with insulin resistance. The inhibition of microRNA expression involved in osteoblast differentiation such as miR-29b, miR-181a, miR-210, and miR-324-3p was also observed. In the study, they also demonstrated the downregulation of β-catenin expression in obese subjects with insulin resistance [27]. β-catenin is involved in the regulation of bone homeostasis by promoting osteoblast activity and suppressing osteoblast turnover [28]. Thus, these results indicated that the obese subjects with insulin resistance may have disturbed osteoblastogenesis via the regulation of Wnt/β-catenin signalling. In line with the aforementioned findings, some other studies have demonstrated that serum sclerostin was significantly higher in individuals with obesity [29] and impaired glucose tolerance [30]. Although patients with T1DM and T2DM with a distinct pathophysiology were associated with a higher expression of Wnt inhibitors, hyperinsulinemia did not affect the serum sclerostin level [29].

In a small-scale study using 20 recruited subjects from a clinical trial, the association between Wnt and insulin signalling was studied among Dutch South Asian men and Dutch white Caucasian individuals with prediabetes who were either overweight or obese. The South Asian men had higher plasma sclerostin, lower Wnt signalling gene expression, and key insulin genes (insulin receptors and glycogen synthase 1) in white adipose tissue compared to the white Caucasians. However, the bone mass and total BMD were similar in both South Asians and white Caucasians [31]. The discrepancy in bone health was not observed in this study because the comparison was conducted among subjects with different ethnicities, albeit with the same medical conditions. Despite the two signalling pathways being influenced by ethnic differences, their results also indicated that the Wnt signalling gene expression was positively associated with insulin gene expression in both ethnicities [31].

Apart from insulin, IGF-1 and its receptors are fundamental in modulating glucose metabolism, insulin production, and skeletal growth [32]. In a study by Ardawi et al., they reported that increased serum sclerostin was positively associated with vertebral fractures among postmenopausal women with T2DM. These changes were accompanied by higher levels of bone resorption markers (such as C-terminal telopeptide (CTX) and NTX) and lower levels of bone formation markers (such as OCN and procollagen 1 intact N-terminal propeptide (P1NP)) among women with vertebral fractures. They also reported that postmenopausal women with T2DM exhibited lower serum levels of IGF-1, indicating a negative association between IGF-1 and vertebral fractures [33]. A previous study determined the relationship between bone turnover markers, Wnt-signalling markers, and IGF-1 levels in healthy boys. This study demonstrated that IGF-1 levels, bone mass, and bone size are increased during skeletal development in puberty [34]. A study by Zhang et al. recruited diabetic osteoporosis patients aged between 66 and 69 years. The results showed that the BMD, bone mineral content (BMC), bone area, T-score, and serum level of 25-hydroxyvitamin D were significantly lower in diabetic osteoporosis patients than in the controls. However, the serum IGF-1 receptor (IGF-1R) was significantly higher when compared to the control group [35].

**Table 1:** The relationship between insulin and the Wnt/β-catenin signalling pathway in human studies.

Type of Study	Subject Characteristics	Findings	Reference
Case-control study	Healthy and T1DM children and adolescents (n = 80)	Insulin and DKK1 were higher but vitamin D, NTX, OCN, and phosphorus levels were lower in T1DM children and adolescents than in the controls.	[24]
Cross-sectional study	T1DM children and adolescents (n = 106; aged 12.2 ± 4 years) and controls (n = 80; aged 11.8 ± 3.4 years)	Higher DKK1 and sclerostin but lower Z-scores were detected in the T1DM subjects than in the controls. Treatment with insulin via continuous subcutaneous infusion improved glycaemic control and Z-scores in the T1DM subjects compared to treatment with insulin via multiple daily injections.	[26]
Cross-sectional study	Obese subjects with (n = 41) and without (n = 41) insulin resistance	Obese subjects with insulin resistance (higher HOMA-IR and lower oral glucose insulin sensitivity index) had higher expressions of DKK1, DKK2, sclerostin, and sFRP-1 but lower expression of osteogenic microRNA and β-catenin.	[27]
Cross-sectional study	Lean (n = 21; aged 25–47 years) and obese (n = 22; aged 27–50 years) women	Insulin sensitivity was inversely associated with serum sclerostin in obese women; hyperinsulinemia did not affect serum sclerostin in both lean and obese women.	[29]
Cross-sectional study	Individuals with normal glucose tolerance (n = 43; aged 44.0 ± 1.9 years) or impaired glucose regulation (n = 79; aged 46.0 ± 1.4 years)	HOMA-IR was positively associated with sclerostin levels; the sclerostin level was higher in individuals with impaired glucose regulation than normal glucose tolerance.	[30]
Clinical trial	Dutch South Asian men with prediabetes who are overweight or obese (n = 10; aged 47 ± 7 years) and Dutch white Caucasians (n = 10; aged 48 ± 6 years)	Wnt signalling gene expression was positively associated with insulin signalling gene expression.	[31]
Cross-sectional study	Postmenopausal women with (n = 482; aged 59.60 ± 7.90 years) and without (n = 482; aged 58.20 ± 6.73 years) T2DM	Serum sclerostin was positively associated but serum IGF-1 was negatively associated with vertebral fractures among postmenopausal women with T2DM.	[33]
Population-based study	Peri-pubertal boys (n = 118; aged 5.1–17.3 years)	IGF-1 level, bone mass, and bone size were increased, reflecting skeletal development in peri-pubertal boys.	[34]
Case-control study	Subjects with (n = 20; aged 69.3 ± 5.3 years) and without (n = 20; aged 66.0 ± 9.0 years) diabetic osteoporosis	Serum IGF-1R level was higher in patients with diabetic osteoporosis compared to the controls.	[35]

Abbreviations: DKK1, Dickkopf-related protein 1; DKK2, Dickkopf-related protein 2; HOMA-IR, homeostatic model assessment of insulin resistance; IGF-1, insulin-like growth factor-1; IGF-1R, insulin-like growth factor-1 receptor; NTX, N-terminal telopeptide; OCN, osteocalcin; sFRP-1, secreted frizzled-related protein-1; T1DM, type 1 diabetes mellitus; T2DM, type 2 diabetes mellitus.

Taken together, the available evidence has demonstrated the influence of insulin secretion, insulin sensitivity, IGF-1 level, and IGF-1R on Wnt signalling molecules. It could be hypothesised that reduced insulin secretion (in T1DM) and impaired insulin sensitivity (in T2DM) are associated with a higher expression of Wnt inhibitors or lower expression of β-catenin, causing impaired bone health in T1DM, T2DM, and/or obese individuals. However, hyperinsulinemia exerts a negligible effect on the expression of Wnt inhibitors. The high level of serum insulin detected in T1DM subjects can be attributed to insulin use as a treatment regimen. On the other hand, the level of IGF-1 increases during skeletal development but reduces in diabetic osteoporotic conditions, reiterating the protective effects of IGF-1 on bone. Although the expression of IGF-1R is high in diabetic osteoporotic conditions, the low level of IGF-1 and high level of sclerostin in serum could be the limiting factors responsible for the higher risk of fractures in humans. In addition, it is recommended that the insulin tolerance test and homeostatic model assessment of insulin resistance (HOMA-IR) should be closely monitored as part of the management of osteoporosis and its related fracture. Proper maintenance of bone health may lead to better glucose and energy metabolism in diabetic patients. Human association studies have confirmed the importance of insulin and IGF-1 in modulating the Wnt-related molecules and inhibitors; thus, targeting these two signalling pathways as therapeutic interventions in human trials recruiting patients with osteoporosis and/or fractures may be the next step of research.

## 6. The Crosstalk between Insulin and Wnt/β-Catenin Signalling in Animal Studies

### 6.1. Streptozotocin (STZ)-Induced Diabetic Animal Model

Several studies have investigated the crosstalk between insulin and Wnt/β-catenin signalling underlying the STZ-induced disturbance in bone homeostasis (Table 2). STZ was used to cause insulin deficiency in rats by damaging the pancreatic β-cells. The STZ-induced diabetes animals had impaired bone quality (indicated by lower bone volume/tissue volume (BV/TV), bone surface (BS), trabecular number (Tb.N), trabecular thickness (Tb.Th), cortical thickness (Cor.Th), calcium level, and BMD), lower compressive strength, decreased osteogenic markers (including alkaline phosphatase (ALP), OCN, type 1 collagen (COL1), osterix (OSX), and distal-less homeobox 5 (Dlx5)), as well as increased osteoclastogenic markers (such as receptor activator of nuclear factor-kappa B ligand (RANKL)) [36,37,38,39]. The mechanism of action underlying the STZ-induced bone loss could be due to a reduction in insulin and IGF-1 levels, leading to the downregulation of phosphorylated Akt (p-Akt), phosphorylated glycogen synthase kinase-3 beta (p-GSK3β), and active β-catenin [36,37]. The activation of β-catenin using 10 mg/kg tamoxifen resulted in increases in bone mass and strength in the trabecular bone of the STZ-induced diabetic mice [36]. Treatment with insulin was able to reverse the detrimental bone changes in the diabetic rats, but such observations were not seen upon IGF-1 treatment [37]. In systemic circulation, IGF-1 binds with IGFBP to stabilise and regulate their action on osteoblasts. Another study suggested that the expression level of IGFBP6 was increased in the STZ-induced diabetic rats [38]. A study by Zhang et al. demonstrated a significant reduction in insulin levels but no change in the phosphorylation of IGF-1R in STZ rats [39]. For other Wnt/β-catenin signalling molecules, the levels of Wnt signalling inhibitors (sclerostin and DKK1) were increased, whereas the levels of Wnt ligands (Wnt3a and Wnt10) were unaltered in STZ-induced diabetes rats [37,38].

### 6.2. Ovariectomised (OVX) Animal Model

Zhang et al. studied the role of the IGF-1R/β-catenin signalling axis in OVX rats and STZ-exacerbating bone impairment in OVX rats (Table 3), suggesting that the potential key role of IGF-1R/β-catenin signalling in the pathogenesis of postmenopausal and/or diabetic osteoporosis. These findings showed that the removal of ovaries and administration of STZ decreased bone strength (indicated by low compressive strength) and impaired bone microarchitecture (indicated by decreased BMD, BV/TV, Tb.N, and Tb.Th) in rats. The OVX rats administered with STZ showed more severe bone damage compared to the OVX rats alone, suggesting that STZ-induced chronic hyperglycaemia acts synergistically with ovariectomy to exert more significant adverse effects on bone. Interestingly, there were increases in the phosphorylation of IGF-1R, GSK3β, and β-catenin, which was in contrast with the fact that GSK3β stimulates the phosphorylation of β-catenin, activating its degradation in the signalling pathway. In addition, PI3K was also activated in the STZ-administered OVX rats [39]. The levels of Wnt ligands and Wnt inhibitors were not evaluated in this study; thus, it remains a challenge to conclude whether the reduced level of β-catenin was attributable to the direct inhibition of the Wnt/β-catenin pathway or indirect action resulted from other signalling pathways. It can also be hypothesised that the increased phosphorylation of GSK3β could be the net outcome from the opposite events resulting from the inhibition of insulin and Wnt/β-catenin signalling as well as the activation of IGF-1 and PI3K pathways.

A low-affinity IGF binder of IGFBP7 may also play an important role in bone metabolism. Zhang et al. reported that a fracture site on tibia rats that were wrapped with a sheet of bone marrow mesenchymal stem cells (BMSCs) overexpressing IGFBP7 had increased bone strength and mineral deposits, evidenced by an increase in BV/TV, Tb.Th, load, stiffness, COL1, and OPG. These findings also demonstrated that IGFBP7 overexpression accelerated bone healing via the activation of the Wnt/β-catenin signalling pathway, as evidenced by the upregulation of the β-catenin level [40].

### 6.3. High-Fat and/or High-Sugar Diet-Induced Animal Model

Using high-fat or high-sugar diets and STZ-induced diabetic rats (Table 4), the alterations of bone microarchitecture (as shown by low calcified nodules, phosphorus, BMC, BMD, BV/TV, Tb.Th, Conn.D, Tb.N, as well as high Tb.Sp and the structure model index (SMI)), bone strength (reduced load, bending strength, and elasticity), osteoclastogenic genes (decreased COL1, Runx2, and OCN), and osteoclastogenic genes (increased CTSK) were noted [35,41,42]. The animals exhibited higher insulin levels, insulin resistance (indicated by increased HOMA-IR), lower IGF-1 levels, and increased IGF-1R phosphorylation [35,41]. Treatment with liraglutide (a glucagon-like peptide-1 analogue; an anti-diabetic agent which has clinical effects on bone metabolism) STZ-induced diabetic mice on a high-fat diet showed restoration in glucose homeostasis, improvement in insulin sensitivity, and enhancement in osteogenic activity via the inhibition of peroxisome proliferator-activated receptor-γ (PPAR-γ) after 4 weeks [43]. The investigation of the underlying mechanism showed the inhibition of β-catenin in the bones of diabetic rats [42]. Studies by Zhang et al. further supported the involvement of Wnt/β-catenin signalling in the development of bone loss in diabetic rats, whereby lower expressions of LRP5 and β-catenin were noted [35,41]. Osteoclastogenesis was also promoted, along with the inhibition of Wnt signalling. High expressions of tumour necrosis factor receptor-associated factor 6 (TRAF6), nuclear factor-kappa B (NF-κB), and the nuclear factor of activated T cells cytoplasmic 1 (NFATc1) were also demonstrated in diabetic rats [41]. The receptor activator of nuclear factor-kappa B (RANK) transmits the RANKL signal to activate the TRAF6 protein and NF-κB, which regulates osteoclast formation by activating the downstream NFATc1 [44]. Moreover, recent in vivo studies have reported the consecutive activation of β-catenin-induced bone loss in differentiated osteoclasts in mice [45]. Thus, it is hypothesised that the induction of osteoclastogenesis might be a direct effect of Wnt activation in osteoclasts or an indirect event resulting from higher RANKL/OPG expression in the osteoblast lineage through the inhibition of the Wnt pathway.

### 6.4. Genetic Animal Model

The crosstalk between insulin and Wnt/β-catenin signalling has also been studied using genetic animal models (Table 5). A study by Lau et al. demonstrated that the conditional disruption of the IGF-1 in osteocytes affects the cross-sectional area and lamellar trabecular bone formation process in response to loading, evidenced by decreased cortical bone area (Cor.Ar), Cor.Th, moment of inertia, BMC, total bone area, and mineral apposition rate (MAR) [46]. Osteocyte-derived IGF-I plays a crucial role in determining the mechanosensitivity of bone. The knockout of IGF-1 in osteocytes abolished the loading-induced activation of the Wnt signalling (evidenced by downregulation of Wnt10b, LRP5, and sFRP-2 but upregulation of sclerostin) and the corresponding osteogenic response [46]. Moreover, IGFBP2 (a conserved family of IGFBP that circulates in the bone marrow microenvironment) rises at peak bone acquisition, indicating its role in skeletal homeostasis. Using the male *Igfbp2^−/−^* mice model, low bone mass accompanied by reduced osteoblast numbers and low bone formation rates were observed and improved when treated with the heparin-binding domain of IGFBP2, as evidenced by a significant increase in bone microstructure and histomorphometry (BV/TV, Tb.Th, Ob.S, and Ob.N). Similarly, the improvement of bone microstructure was also detected in female *Igfbp2^−/−^* mice treated with the heparin-binding domain of IGFBP2. These findings demonstrated that the heparin-binding domain of IGFBP2 had an anabolic effect on the skeleton [47].

The knockdown or mutation of LRP4/5 that is resistant to sclerostin leads to dramatic changes in bone phenotype, glucose, and insulin homeostasis. A study by Kim et al. demonstrated that LRP4 expression in adipocytes and osteoblasts differentially impacts sclerostin functions, glucose, and insulin homeostasis. Lrp4 deficiency in the adipocytes of mice reduced sclerostin levels but improved glucose and insulin tolerance. However, the loss of LRP4 function in the osteoblasts of mice showed increased sclerostin levels, impaired glucose tolerance, and insulin sensitivity [48]. In a different animal model of LRP5-mutant mice fed with a high-fat diet, there were decreases in BV/TV and uncarboxylated OCN production along with increases in serum glucose and insulin levels after fasting, suggesting the occurrence of bone loss and insulin resistance. The inhibition of Akt (evidenced by reduced phosphorylated Akt) was also noted in the LRP5 mutant mice fed with a high-fat diet. These findings suggested that high-fat diet feeding caused deficient OCN production in mice lacking LRP5, leading to disturbances in glucose metabolism, insulin sensitivity, and skeletal homeostasis [49]. Another study suggested that the development, proliferation, and survival of pancreatic β-cells, as well as the β-cell compensation for peripheral insulin resistance, are mediated by the IGF-1R signalling pathway through IRS-2. The expression of the insulin receptor, IGF-1R, and IRS-2 in the islets was markedly decreased, which was consistent with the impaired glucose-induced insulin secretion in LRP5-deficient mice fed with a high-fat diet. These findings reiterated that LRP5 maintains normal cell function via the transcriptional regulation of insulin receptors, IGF-1R, and IRS-2 [50].

The gain-of-function of LRP5 co-receptors which are resistant towards sclerostin is associated with high bone mass. Using a sclerostin-resistant, LRP5-mutant (gain-of-function) model, there were significant increases in the volumetric trabecular and cortical regions of bone (indicated by higher BMD, BV/TV, Tb.Th, Cor.Th, and Cor.Ar, but lower Tb.Sp), mechanical strength (reflected by higher load and stiffness), and OCN levels [51]. Similarly, mice with LRP5-mutant (gain-of-function) and insulin-dependent spontaneous hyperglycaemia displayed better bone health with increased insulin sensitivity [51]. Sclerostin binds to LRP5 and antagonize the Wnt canonical signalling. Therefore, the results of mouse genetic models showed that Wnt signalling has a favourable effect on glucose metabolism.

Overall, the in vivo findings suggested that the perturbation of insulin homeostasis influences the expression of Wnt/β-catenin signalling molecules and vice versa. Despite the difference in the pathophysiology between T1DM and T2DM, the deterioration of bone health through inhibition of the canonical Wnt/β-catenin signalling has been reported. On the other hand, the loss of function in LRP5 receptors in bone is associated with impaired insulin sensitivity, which can be reversed by the gain-of-function. Apart from the well-known positive action of Wnt protein on bone formation, recent evidence indicates that insulin/IGF-1-activated Wnt signalling suppresses osteoclast proliferation, differentiation, and maturation.

## 7. The Crosstalk between Insulin and Wnt/β-Catenin Signalling Molecules in Cell Culture Models

Several in vitro studies using osteoblasts, mesenchymal stem cells, preosteoblasts (Table 6), or adipocytes (Table 7) have demonstrated the crosstalk between the signalling networks of insulin and IGF-1 with Wnt/β-catenin signalling that is responsible for bone growth and maintenance. In an earlier study, the upregulation of IGF-1 and Wnt signalling molecules was observed in primary osteoblasts isolated from C57BL/6J mice and subjected to fluid flow shear stress that represents a model of mechanical loading. Several key molecules including Wnt1, Wnt3a, Wnt5a, LRP5, β-catenin, LEF-1, and Axin in the Wnt pathway, as well as IGF-1 and Fos proto-oncogene (c-Fos) in IGF-1 signalling, were upregulated [52]. A study by Sunters et al. investigated the response of osteoblast-like (UMR-106) cells subjected to dynamic strain after incubation with 50 ng/mL of IGF-1. The findings showed that the β-catenin was activated by the activation of IGF-1R following the addition of IGF-1, subsequently causing the PI3K-mediated activation of Akt and the increased phosphorylation of GSK3β [53]. Current evidence has confirmed the crosstalk between IGF-1 and Wnt/β-catenin in osteoblasts subjected to mechanical strain, but the crosstalk in osteocytes awaits further clarification. Osteocytes are the main source of sclerostin and DKK1 expression influenced by mechanobiology. Theoretically, the ability of the resident cells, mainly the osteoblast and osteocytes, to appropriately respond to mechanical strain would determine the capacity of the bones to adjust their mass and architecture in favour of greater bone strength to withstand forces and mechanical loads [54]. Based on the aforementioned evidence, the crosstalk between insulin/IGF-1 and Wnt/β-catenin signalling molecules in osteocytes is worth investigating because osteocytes may be an essential coordinator for bone modelling and remodelling upon mechanical loading.

The role of IGFBP-2 in increasing bone mass was thought to be partly mediated by the activity of its heparin-binding domain. The heparin-binding domain of IGFBP-2 enhanced cell proliferation, mineralisation, and osteogenesis in calvarial osteoblasts and bone marrow stromal cells in *Igfbp2^−/−^* mice, as measured by increased ALP, Runx2, and OCN expression. The cellular actions involved were evident by the activation of the Akt and cytosolic accumulation of β-catenin in response to the heparin-binding domain of the IGFBP-2 treatment. These findings reiterated that the heparin-binding domain of IGFBP-2 exerted anabolic activity by activating the IGF-1/Akt and Wnt/β-catenin signalling pathways [47]. In another study, Zhang et al. demonstrated the role of IGFBP-7 in the osteogenic differentiation of human bone marrow-derived mesenchymal stem cells obtained from male patients with femoral or tibial fractures. The study found that IGFBP-7 overexpression promoted osteogenic differentiation, which was, in part, mediated through the Wnt/β-catenin signalling pathway. Meanwhile, IGFBP7 knockdown decreased cell proliferation and reduced the level of osteo-specific gene expression, ALP activity, and calcium deposit formation [40].

The role of insulin and IGF-1 in promoting cell proliferation and osteogenic differentiation via the Wnt/β-catenin pathway was also studied in knockdown cells. The murine preosteoblastic cell line (MC3T3-E1) was transfected with the small interfering RNA (siRNA) of the insulin receptor to represent an insulin receptor-knockdown model. Lower levels of OSX, OCN, and OPG but higher levels of Runx2 and RANKL were noted in the knockdown model compared to the control, demonstrating lower osteoblastic but higher osteoclastic activities [38]. In another study, the bone marrow-derived mesenchymal stem cells were transfected with siRNA of Wnt3a to mimic a Wnt3a-knockdown model and treated with IGF-1. The knockdown cells treated with IGF-1 exhibited lower cell proliferation, mineralization, and osteogenic expression compared to the normal cells treated with IGF-1. The levels of β-catenin and cyclin D1 were also downregulated in the IGF-1-treated Wnt3a-knockdown cells compared to the IGF-1-treated normal cells. The results suggested that the ability of IGF-1 to promote osteogenic differentiation was decreased after the Wnt/β-catenin pathway was inhibited [55].

Mesenchymal stem cells are multipotent stem cells that serve as a progenitor with a balanced differentiation commitment to a variety of cells, including osteoblasts, chondrocytes, and adipocytes. The dedifferentiation of mature murine adipocytes and insulin resistance were induced upon Wnt activation by Wnt3a. The presence of Wnt3a stimulated osteogenesis in the dedifferentiated adipocytes, whereby BMP4, Runx2, and mineralisation were increased. Mechanistically, there were marked decreases in the basal and insulin-stimulated glucose transport, phosphorylation of the insulin receptor, IRS-1, and Akt. For Wnt-signalling molecules, the level of active β-catenin was increased but the DKK1 level was unchanged [56]. A recent study investigated the involvement of the Wnt/GSK-3β/β-catenin pathway using glucose-stimulated human adipocyte-derived mesenchymal stem cells, mimicking the environment of diabetes mellitus. High glucose induced osteogenic differentiation but suppressed adipogenic differentiation, which was reversed upon treatment with liraglutide. Specifically, it was found that liraglutide suppressed adipogenesis-related genes (CCAAT enhancer-binding protein alpha (C/EBP-α) and PPAR-γ) but promoted the osteogenic differentiation genes’ (COL1 and OCN) expression in human adipocyte-derived mesenchymal stem cells cultured under high glucose conditions. Upon liraglutide treatment, GSK3β was inhibited and subsequently upregulated β-catenin [43].

Taken together, the in vitro findings provide an insight into the role of IGF-1 and IGFBP in the activation of the Wnt/β-catenin pathway in bone-forming processes with or without mechanical stress. The potential crosstalk between the two signalling pathways became apparent with the use of loss-of-function mutation models. The osteogenic proliferation and differentiation capabilities of IGF-1 are attenuated without a functional Wnt ligand to stabilise β-catenin. Osteogenesis is inhibited, whereas osteoclastogenesis is promoted in osteoblasts lacking insulin receptors. Apart from its effect on bone formation and resorption activities, the presence of Wnt ligands induces the transdifferentiation from adipogenic to osteogenic lineage.

## 8. Perspectives

**Figure 4:**
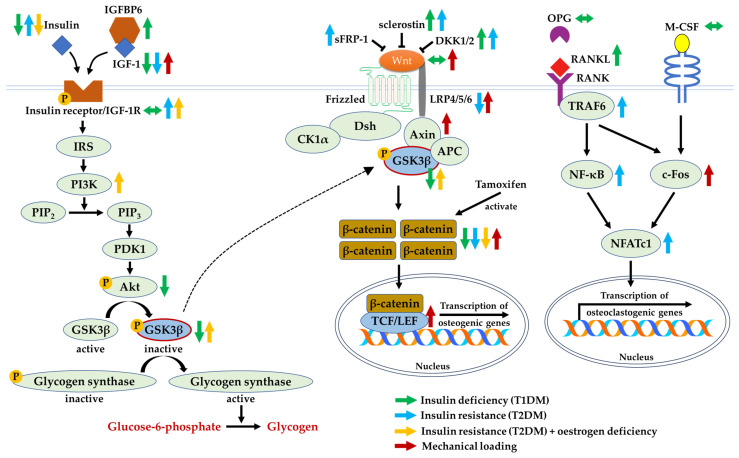
The crosstalk between insulin/IGF-1 and Wnt/β-catenin signalling molecules during insulin deficiency, insulin resistance, oestrogen deficiency, or subjected to mechanical loading. Arrow pointing upward (↑) indicates an increase in expression, arrow pointing downward (↓) indicates a decrease in expression, whereas the arrow pointing both sides (↔) indicates no change in expression. Abbreviations: Akt, protein kinase B; APC, adenomatosis polyposis coli; Axin, axis inhibition protein; c-Fos, Fos proto-oncogene; CK1α, casein kinase 1α; DKK1/2, Dickkopf-related protein 1 or 2; Dsh, phosphoprotein Dishevelled; GSK3β, glycogen synthase kinase-3 beta; IGF-1, insulin-like growth factor-1; IGF-1R, insulin-like growth factor-1 receptor; IGFBP6, insulin-like growth factor binding protein 6; IRS, insulin receptor substrate; LEF, lymphoid enhancing factor; LRP4/5/6, lipoprotein receptor-related protein 4/5/6; NFATc1, nuclear factor of activated T cells cytoplasmic 1; NF-κB, nuclear factor-kappa B; OPG, osteoprotegerin; p-Akt, phosphorylated protein kinase B; PDK1, phosphoinositide-dependent kinase-1; PI3K, phosphoinositide-3-kinases; p-glycogen synthase, phosphorylated glycogen synthase; p-GSK3β, phosphorylated glycogen synthase kinase-3 beta; p-IGF-1R, phosphorylated insulin-like growth factor-1 receptor; p-insulin receptor, phosphorylated insulin receptor; PIP_2_, phosphatidylinositol-4,5-biphosphate; PIP_3_, phosphatidylinositol-3,4,5-triphosphate; RANK, receptor activator of nuclear factor-kappa B; RANKL, receptor activator of nuclear factor-kappa B ligand; sFRP-1, secreted frizzled-related protein-1; T1DM, type 1 diabetes mellitus; T2DM, type 2 diabetes mellitus; TCF, T-cell factor; TRAF6, tumour necrosis factor receptor-associated factor 6; Wnt, Wingless ligand.

The presence of insulin and IGF-1 activates a series of downstream signalling cascades that cause the activation of Akt and inhibition of GSK3β. Similarly, GSK3β is inhibited in the presence of Wnt agonists. The phosphorylated GSK3β activates glycogen synthase and causes an accumulation of β-catenin, reiterating its multifaceted role in improving insulin homeostasis and bone health concurrently. Based on the evidence, GSK3β may appear as a potential point of convergence between the two signalling pathways involved. The possible crosstalk between insulin/IGF-1 and Wnt/β-catenin signalling molecules has been clearly elucidated (Figure 4).

Insulin deficiency in T1DM and non-functional insulin in T2DM are associated with higher expressions of Wnt inhibitors, leading to the suppression of the Wnt/β-catenin pathway. Hence, osteoblast differentiation, as the compensatory mechanism for bone resorption, could be attenuated. The investigation of its underlying mechanism in a controlled experiment setting using in vivo and in vitro models demonstrated that low levels of insulin and IGF-1 inhibited Akt and subsequently activated GSK3β, causing the degradation of β-catenin. Although insulin deficiency and insulin resistance are implicated in the development and progression of bone loss, studies elucidating the effects of insulin treatment on bone health yielded heterogeneous outcomes, either having beneficial or negligible effects [26,37]. Hence, correcting impaired insulin sensitivity and glucose tolerance may be a more viable treatment method compared to administering insulin to improve bone health. Furthermore, the possible crosstalk between the insulin/IGF-1 and Wnt/β-catenin pathways has been validated using knockout models. The deficiency of IGF-1 suppresses the Wnt signalling pathway. Moreover, the lack-of-function of LRP5 causes insulin resistance, which can be reversed using the gain-of-function.

The limitations of the documented evidence are acknowledged. Firstly, previous investigations have focused on the involvement of insulin-, IGF-1- and Wnt-related signalling molecules in osteoblasts, with a paucity of studies reporting on osteoclasts and osteocytes. This could be due to the fact that canonical Wnt/β-catenin are the most implicated transduction network in osteoblastic differentiation and bone formation. Zhang et al. reported the activation of RANK/RANKL/OPG-mediated osteoclastogenesis with a reduced accumulation of active β-catenin in a diabetic model, suggesting the possible direct or indirect interaction between insulin and Wnt/β-catenin cascades in modulating bone resorption [41]. However, the crosstalk in the aspect of osteoclastogenesis has not been fully understood. Secondly, there is a lack of in vitro studies on the relationship between insulin and Wnt signalling, with more effort being given to the effects of IGF-1 and Wnt molecules, which warrant further investigation. At this juncture, several research gaps need to be filled by investigators. The use of the GSK3β-knockdown model may be the future direction of research to investigate the role of GSK3β inhibition as a therapeutic target in insulin disturbance and bone-related disorders. The crosstalk between insulin/IGF-1 and Wnt signalling pathways in osteocytes is also pivotal in understanding how insulin homeostasis and IGF-1 levels affect the expression of sclerostin and DKK1. A strength of the current review is its focus on the association between the insulin and Wnt signalling pathways to provide an overview of the direct outcomes of insulin/IGF-1 on Wnt-related components as well as the direct effects of Wnt proteins/inhibitors on insulin sensitivity. Glucose metabolism and other signalling pathways are not the current topic of discussion, which may be a limitation of the current review.

## 9. Conclusions

The inactivation of insulin or IGF-1 signalling results in the suppression of the Wnt/β-catenin pathway and eventually inhibits osteoblastogenesis. Low levels of insulin or IGF-1 and insulin resistance cause the inhibition of Akt phosphorylation and subsequent reductions in phosphorylated GSK3β, resulting in the degradation of β-catenin proteins. Hence, GSK3β may emerge as a molecular link between the insulin and Wnt/β-catenin pathways in orchestrating both skeletal and insulin homeostasis.

## Figures and Tables

**Table 2:** The relationship between insulin and the Wnt/β-catenin signalling pathway in STZ-induced diabetic animal models.

Type of Animal Model	Treatment/Intervention (Dose, Route, and Duration)	Findings	Reference
STZ-induced diabetic mice	-	BV/TV: ↓, Tb.Th: ↓, Tb.N: ↓, Tb.Sp: ↑, BA/TA: ↓, Cor.Th: ↓, Ob.N: ↓, Ob.S: ↓, BFR: ↓, ALP: ↓, OSX: ↓, IGF-1R: ↓, LEF-1: ↓, TCF: ↓, Axin: ↓, p-Akt: ↓, p-GSK3β: ↓, β-catenin: ↓, DKK1: ↑	[36]
	Tamoxifen (10 mg/kg, i.p., 4 days)	BV/TV: ↑, Tb.Th: ↑, BA/TA: ↔, Cor.Th: ↔, mineralisation: ↑, stiffness: ↑, force: ↑, Ob.N: ↑, Ob.S: ↑, Oc.N: ↓, Oc.S: ↓, BFR: ↑, Runx2: ↑, OSX: ↑, OCN: ↔, OPG: ↑, RANKL: ↑	
STZ-induced diabetic rats	-	Insulin: ↓, IGF-1 (serum): ↓, IGF-1 (tibia): ↓, IGF-1R: ↓, TRAP: ↔, CTSK: ↔, calcium: ↓, hydroxyproline: ↓, urine deoxypyridinoline: ↔, BV/TV: ↓, BS: ↓, Tb.Th: ↓, Tb.N: ↓, Ob.N: ↓, Oc.N: ↔, BMP-2: ↔, Dlx5: ↓, Runx2: ↓, OSX: ↓, ALP: ↓, OCN: ↓, COL1: ↓, Wnt3a: ↔, LRP5: ↔, β-catenin: ↓, p-GSK3β: ↓, p-Akt: ↓, sclerostin: ↑, DKK1: ↑	[37]
	Bovine insulin (1.6 U/day, continuous infusion for 4 weeks)	Insulin: ↑, IGF-1 (serum): ↑, IGF-1 (tibia): ↑, IGF-1R: ↑, TRAP: ↔, CTSK: ↔, calcium: ↑, hydroxyproline: ↑, urine deoxypyridinoline: ↔, BV/TV: ↑, BS: ↑, Tb.Th: ↑, Tb.N: ↑, Ob.N: ↑, Oc.N: ↔, BMP-2: ↔, Dlx5: ↑, Runx2: ↑, OSX: ↑, ALP: ↑, OCN: ↑, COL1: ↑, Wnt3a: ↔, LRP5: ↔, β-catenin: ↑, p-GSK3β: ↑, p-Akt: ↑, sclerostin: ↓, DKK1: ↓	
	Human recombinant IGF-1 (50 ng/day, continuous infusion for 4 weeks)	Insulin: ↔, IGF-1 (serum): ↑, IGF-1 (tibia): ↔, IGF-1R: ↔, TRAP: ↔, CTSK: ↔, calcium: ↔, hydroxyproline: ↔, urine deoxypyridinoline: ↔, BV/TV: ↔, BS: ↔, Tb.Th: ↔, Tb.N: ↔, Ob.N: ↔, Oc.N: ↔, BMP-2: ↔, Dlx5: ↔, Runx2: ↔, OSX: ↔, ALP: ↔, OCN: ↔, COL1: ↔, Wnt3a: ↔, LRP5: ↔, β-catenin: ↔, p-GSK3β: ↔, p-Akt: ↔, sclerostin: ↔, DKK1: ↔	
STZ-induced diabetic rats	-	ALP: ↓, OCN: ↓, Runx2: ↔, M-CSF: ↔, OPG: ↔, RANKL: ↑, CTSK: ↔, TRAP: ↔, MMP-9: ↓, insulin receptor: ↔, IGF-1: ↔, IGFBP4: ↔, IGFBP6: ↑, Wnt10: ↔, β-catenin: ↔	[38]
STZ-induced diabetic rats	-	Insulin: ↓, BMD: ↓, compressive strength: ↓, BV/TV: ↓, Tb.N: ↓, Tb.Th: ↓, Tb.Sp: ↑, BS/BV: ↑, OCN: ↓, p-IGF-1R: ↔, p-GSK3β: ↑, p-β-catenin: ↔, PI3K: ↔	[39]

Abbreviations: ALP, alkaline phosphatase; Axin, axis inhibition protein; BA/TA, bone area/tissue area; BFR, bone formation rate; BMD, bone mineral density; BMP-2, bone morphogenetic protein-2, BS, bone surface; BS/BV, bone surface/bone volume; BV/TV, bone volume/total volume, COL1, type 1 collagen; Cor.Th, cortical thickness; CTSK, cathepsin K; DKK1, Dickkopf-related protein 1; Dlx5, distal-less homeobox 5; IGF-1, insulin-like growth factor-1; IGF-1R, insulin-like growth factor-1 receptor; IGFBP4, insulin-like growth factor binding protein 4; IGFBP6, insulin-like growth factor binding protein 6; LEF-1, lymphoid enhancing factor-1; LRP5, lipoprotein receptor-related protein 5; M-CSF, macrophage colony-stimulating factor; Ob.N, osteoblast number; Ob.S, osteoblast surface; Oc.N, osteoclast number; Oc.S, osteoclast surface; OCN, osteocalcin; OPG, osteoprotegerin; OSX, osterix; p-Akt, phosphorylated protein kinase B; p-β-catenin, phosphorylated β-catenin; p-GSK3β, phosphorylated glycogen synthase kinase-3 beta; p-IGF-1R, phosphorylated insulin-like growth factor-1 receptor; PI3K, phosphoinositide-3-kinases; RANKL, receptor activator of nuclear factor-kappa B ligand; Runx2, Runt-related transcription factor 2; STZ, streptozotocin; Tb.N, trabecular number; Tb.Sp, trabecular separation; Tb.Th, trabecular thickness; TCF, T-cell factor; TRAP, tartrate-resistant acid phosphatase; Wnt10, Wnt family member 10; Wnt3a, Wnt family member 3A; ↑, increase; ↓, decrease; ↔, no change.

**Table 3:** The relationship between insulin and the Wnt/β-catenin signalling pathway in OVX and fractured animal models.

Type of Animal Model	Treatment/Intervention (Dose, Route, and Duration)	Findings	Reference
OVX rats	-	Insulin: ↔, BMD: ↓, compressive strength: ↓, BV/TV: ↓, Tb.N: ↓, Tb.Th: ↓, Tb.Sp: ↔, BS/BV: ↔, OCN: ↑, p-IGF-1R: ↔, p-GSK3β: ↔, p-β-catenin: ↔, PI3K: ↑	[39]
OVX- and STZ-induced diabetic osteoporotic rats		Insulin: ↓, BMD: ↓, compressive strength: ↓, BV/TV: ↓, Tb.N: ↓, Tb.Th: ↓, Tb.Sp: ↑, BS/BV: ↑, OCN: ↓, p-IGF-1R: ↑, p-GSK3β: ↑, p-β-catenin: ↑, PI3K: ↑	
Tibial fractured rats	BMSCs overexpressing IGFBP7	BV/TV: ↑, Tb.Th: ↑, load: ↑, stiffness: ↑, defects were bridged with cortical bone, COL1: ↑, OPG: ↑, β-catenin: ↑	[40]

Abbreviations: BMD, bone mineral density; BMSCs, bone marrow mesenchymal stem cells; BS/BV, bone surface/bone volume; BV/TV, bone volume/total volume, COL1, type 1 collagen; IGFBP7, insulin-like growth factor binding protein 7; OCN, osteocalcin; OPG, osteoprotegerin; OVX, ovariectomised; p-β-catenin, phosphorylated β-catenin; p-GSK3β, phosphorylated glycogen synthase kinase-3 beta; p-IGF-1R, phosphorylated insulin-like growth factor-1 receptor; PI3K, phosphoinositide-3-kinases; STZ, streptozotocin; Tb.N, trabecular number; Tb.Sp, trabecular separation; Tb.Th, trabecular thickness; ↑, increase; ↓, decrease; ↔, no change.

**Table 4:** The relationship between insulin and the Wnt/β-catenin signalling pathway in high-fat and/or high-sugar diet-induced animal models.

Type of Animal Model	Treatment/Intervention (Dose, Route, and Duration)	Findings	Reference
High-fat/sugar diet- and STZ-induced diabetic rats	-	BMD: ↓, BMC: ↓, compressive strength: ↓, BV/TV: ↓, Tb.Th: ↓, BS/BV: ↓, IGF-1R: ↑, p-IGF-1R: ↑, p-GSK3β: ↑, p-β-catenin: ↑	[35]
High-fat/sugar diet- and STZ-induced diabetic rats	-	Insulin: ↑, HOMA-IR: ↑, calcium: ↔, phosphorus: ↓, ALP: ↑, BMD: ↓, Runx2: ↓, LRP5: ↓, β-catenin: ↓, TRAF6: ↑, NF-κB: ↑, NFATc1: ↑	[41]
High-fat diet- and STZ-induced diabetic rats	-	Calcified nodules: ↓, load: ↓, bending strength: ↓, elastic modulus: ↓, BV/TV: ↓, Tb.Th: ↓, Conn.D: ↓, Tb.N: ↓, Tb.Sp: ↑, SMI: ↑, CTSK: ↑, IGF-1: ↓, β-catenin: ↓, p-β-catenin: ↑	[42]
High-fat diet- and STZ-induced diabetic mice	Liraglutide (0.6 mg/kg/day, i.p., 4 weeks)	Insulin sensitivity: ↑, insulin tolerance: ↑, COL1: ↑, Runx2: ↑, OCN: ↑	[43]

Abbreviations: ALP, alkaline phosphatase; BMC, bone mineral content; BMD, bone mineral density; BS/BV, bone surface/bone volume; BV/TV, bone volume/total volume, COL1, type 1 collagen; Conn.D, connectivity density; CTSK, cathepsin K; HOMA-IR, homeostatic model assessment of insulin resistance; IGF-1R, insulin-like growth factor-1 receptor; i.p., intraperitoneal; LRP5, lipoprotein receptor-related protein 5; NFATc1, nuclear factor of activated T cells cytoplasmic 1; NF-κB, nuclear factor-kappa B; OCN, osteocalcin; p-β-catenin, phosphorylated β-catenin; p-GSK3β, phosphorylated glycogen synthase kinase-3 beta; p-IGF-1R, phosphorylated insulin-like growth factor-1 receptor; Runx2, Runt-related transcription factor 2; SMI, structure model index; STZ, streptozotocin; Tb.N, trabecular number; Tb.Sp, trabecular separation; Tb.Th, trabecular thickness; TRAF6, tumour necrosis factor receptor-associated factor 6; ↑, increase; ↓, decrease; ↔, no change.

**Table 5:** The relationship between insulin and the Wnt/β-catenin signalling pathway in genetic animal models.

Type of Animal Model	Treatment/Intervention (Dose, Route, and Duration)	Findings	Reference
Osteocyte IGF-1 conditional knockout mice subjected to four-point bending	-	Cor.Ar: ↓, Cor.Th: ↓, moment of inertia: ↓, BMD: ↔, BMC: ↓, total bone area: ↓, BV/TV: ↔, MAR: ↓, BFR: ↔, c-Fos: ↓, IGF-1: ↓, Runx2: ↓, OCN: ↓, Wnt10b: ↓, sclerostin: ↑, LRP5: ↓, sFRP-2: ↓, DKK1: ↔, β-catenin: ↓	[46]
Male *Igfbp2^−/−^* mice	-	BV/TV: ↓, Tb.N: ↓, Tb.Th: ↔, Tb.Sp: ↑	[47]
	Heparin-binding domain of IGFBP2 (50 μg, 5 times/week for 3 weeks)	BV/TV: ↑, Tb.N: ↔, Tb.Th: ↑, Tb.Sp: ↔, osteoid surface: ↔, Ob.S: ↑, Ob.N: ↑, ES: ↔, Oc.S: ↔, Oc.N: ↔, MAR: ↔, BFR: ↔	
Female *Igfbp2^−/−^* mice	Heparin-binding domain of IGFBP2 (50 μg, 5 times/week for 3 weeks)	BV/TV: ↑, Tb.N: ↑, Tb.Th: ↔, Tb.Sp: ↓	
LRP4-deficient mice	-	Sclerostin: ↑, insulin sensitivity: ↓, OCN: ↑	[48]
LRP5-deficient mice fed with high-fat diet	-	BV/TV: ↓, insulin: ↑, glucose: ↑, insulin resistance, OCN: ↓, p-Akt: ↓	[49]
LRP5-deficient mice fed with high-fat diet	-	Insulin secretion: ↓, IGF-1: ↓, IRS-2: ↓, insulin receptor: ↓	[50]
Sclerostin-resistant LRP5-mutant (gain-of-function) mice	-	BMD: ↑, BV/TV: ↑, Tb.Th: ↑, Tb.Sp: ↓, Cor.Th: ↑, Cor.Ar: ↑, load: ↑, stiffness: ↑, OCN: ↑	[51]
Sclerostin-resistant LRP5-mutant (gain-of-function) and insulin-dependent diabetic mice	-	BMD: ↑, BV/TV: ↑, Tb.Th: ↑, Tb.Sp: ↓, Cor.Th: ↑, Cor.Ar: ↑, load: ↑, stiffness: ↑, insulin sensitivity: ↑, OCN: ↔	

Abbreviations: BFR, bone formation rate; BMC, bone mineral content; BMD, bone mineral density; BV/TV, bone volume/total volume, c-Fos, Fos proto-oncogene; Cor.Ar, cortical area; Cor.Th, cortical thickness; DKK1, Dickkopf-related protein 1; IGF-1, insulin-like growth factor-1; IGFBP2, insulin-like growth factor binding protein 2; IRS-2, insulin-receptor substrate-2; LRP4, lipoprotein receptor-related protein 4; LRP5, lipoprotein receptor-related protein 5; MAR, mineral apposition rate; Ob.N, osteoblast number; Ob.S, osteoblast surface; Oc.N, osteoclast number; Oc.S, osteoclast surface; OCN, osteocalcin; p-Akt, phosphorylated protein kinase B; p-IGF-1R, phosphorylated insulin-like growth factor-1 receptor; Runx2, Runt-related transcription factor 2; sFRP-2, secreted frizzled-related protein-2; Tb.N, trabecular number; Tb.Sp, trabecular separation; Tb.Th, trabecular thickness; Wnt10b, Wnt family member 10b; ↑, increase; ↓, decrease; ↔, no change.

**Table 6:** The relationship between insulin and the Wnt/β-catenin signalling pathway in in vitro studies using osteoblasts, mesenchymal stem cells, or preosteoblasts.

Type of Cells	Treatment/Intervention (Concentration)	Findings	Reference
Primary osteoblasts isolated from C57BL/6J mice subjected to fluid shear	-	IGF-1: ↑, c-Fos: ↑, Wnt1: ↑, Wnt3a: ↑, Wnt5a: ↑, LRP5: ↑, β-catenin: ↑, LEF-1: ↑, Axin: ↑	[52]
Osteoblast-like (UMR-106) cells subjected to dynamic strain	IGF-1 (50 ng/mL)	p-Akt: ↑, p-GSK3β: ↑, β-catenin: ↑	[53]
Calvarial osteoblasts and bone marrow stromal cells isolated from *Igfbp2^−/−^* mice	Heparin-binding domain of IGFBP2 (2 μg/mL)	Cell proliferation: ↑, mineralisation: ↑, ALP: ↑, Runx2: ↑, OCN: ↑, p-Akt: ↑, β-catenin: ↑	[47]
Bone marrow-derived mesenchymal stem cells isolated from male patients with femoral or tibial fractures	IGFBP7 overexpression	Cell proliferation: ↔, ALP: ↑, Runx2: ↑, OCN: ↑, COL1: ↑, OSX: ↑, calcium deposits: ↑, p-β-catenin: ↑, p-Akt: ↔, p-ERK: ↔, p-p38: ↔, p-JNK: ↔	[40]
	IGFBP7 knockdown	Cell proliferation: ↓, ALP: ↓, Runx2: ↓, OCN: ↓, COL1: ↓, OSX: ↓, calcium deposits: ↓, p-β-catenin: ↓, p-Akt: ↔, p-ERK: ↔, p-p38 MAPK: ↔, p-JNK: ↔	
Insulin receptor-knockdown MC3T3-E1 cells	-	Insulin receptor: ↓, IRS-1: ↔, Runx2: ↑, OSX: ↓, OCN: ↓, OPG: ↓, RANKL: ↑	[38]
Wnt3a-knockdown bone marrow-derived mesenchymal stem cells	IGF-1 (80 ng/mL)	Cell proliferation: ↓, β-catenin: ↓, cyclin D1: ↓, Runx2: ↓, OPN: ↓	[55]

Abbreviations: ALP, alkaline phosphatase; Axin, axis inhibition protein 2; COL1, type 1 collagen; IGFBP2, insulin-like growth factor binding protein 2; IGFBP7, insulin-like growth factor binding protein 7; IGF-1, insulin-like growth factor-1; IRS-1, insulin receptor substrate-1; LEF-1, lymphoid enhancing factor-1; LRP5, lipoprotein receptor-related protein 5; MC3T3-E1, murine preosteoblastic cell line; OCN, osteocalcin; OPG, osteoprotegerin; OPN, osteopontin; OSX, osterix; p-Akt, phosphorylated protein kinase B; p-β-catenin, phosphorylated β-catenin; p-ERK, phosphorylated extracellular signal-regulated kinase; p-GSK3β, phosphorylated glycogen synthase kinase-3 beta; p-JNK, phosphorylated c-Jun N-terminal kinase; p-p38 MAPK, phosphorylated p38 mitogen-activated protein kinase; RANKL, receptor activator of nuclear factor-kappa B ligand; Runx2, Runt-related transcription factor 2; Wnt3a, Wnt family member 3a; Wnt5a, Wnt family member 5a; ↑, increase; ↓, decrease; ↔, no change.

**Table 7:** The relationship between insulin and the Wnt/β-catenin signalling pathway in in vitro studies using adipocytes or adipocyte-derived cells.

Type of Cells	Treatment/Intervention (Concentration)	Findings	Reference
Murine mature adipocytes	Wnt3a (50 ng/mL)	p-insulin receptor: ↓, p-IRS-1: ↓, p-Akt: ↓, BMP-4: ↑, Runx2: ↑, mineralisation: ↑, β-catenin: ↑, LRP6: ↓, Axin: ↔, DKK1: ↔	[56]
High glucose-stimulated human adipose-derived mesenchymal stem cells	Liraglutide (100 nM)	COL1: ↑, Runx2: ↔, OCN: ↑, C/EBP-α: ↓, PPAR-γ: ↓, GSK3β: ↓, p-GSK3β: ↑, β-catenin: ↑	[43]

Abbreviations: Axin, axis inhibition protein 2; BMP-4, bone morphogenetic protein-4; C/EBP-α, CCAAT enhancer-binding protein alpha; COL1, type 1 collagen; DKK1, Dickkopf-related protein 1; GSK3β, glycogen synthase kinase-3 beta; LRP6, lipoprotein receptor-related protein 6; OCN, osteocalcin; p-Akt, phosphorylated protein kinase B; p-GSK3β, phosphorylated glycogen synthase kinase-3 beta; p-insulin receptor, phosphorylated insulin receptor; p-IRS-1, phosphorylated insulin receptor substrate-1; PPAR-γ, peroxisome proliferator-activated receptor-γ; Runx2, Runt-related transcription factor 2; Wnt3a, Wnt family member 3a; ↑, increase; ↓, decrease; ↔, no change.

## Data Availability

Not applicable.
